# Antiviral Activity of Glucosyl Hesperidin Against Feline Calicivirus

**DOI:** 10.3390/microorganisms13102332

**Published:** 2025-10-10

**Authors:** Sung-Sook Choi, Sun-Hyung Lee, Kyung-Ae Lee

**Affiliations:** 1 Department of Food and Nutrition, Duksung Women’s University, Seoul 01370, Republic of Korea; choiss@duksung.ac.kr; 2 Department of Food and Nutrition, Korea National Open University, Seoul 03087, Republic of Korea; yjbio21@naver.com; 3 Department of Food and Nutrition, Anyang University, Anyang 14028, Republic of Korea

**Keywords:** norovirus, feline calicivirus, flavonoid, glucosyl hesperidin, antiviral activity

## Abstract

The objective of this study was to evaluate the antiviral activity of glucosyl hesperidin (GH), a water-soluble derivative of hesperidin with known antioxidant and anti-inflammatory properties, in order to explore its potential applications. Antiviral activity was assessed using feline calicivirus (FCV), a surrogate model for human norovirus, a major foodborne pathogen. Cytotoxicity testing in Crandell–Rees feline kidney (CRFK) cells demonstrated that GH exhibited high biocompatibility, maintaining 100% cell viability at concentrations up to 8000 μM. Antiviral efficacy assays revealed that GH inhibited FCV replication in a concentration-dependent manner across the range of 250~8000 μM, with a half-maximal inhibitory concentration (IC_50_) of 3281 μM. Complete viral inhibition, however, was not achieved at the maximum concentration tested. In conclusion, GH was shown to inhibit FCV while maintaining low cytotoxicity, indicating its potential as a natural, water-soluble candidate for the suppression of norovirus.

## 1. Introduction

Norovirus, a single-stranded RNA virus belonging to the family Caliciviridae, is a leading cause of acute gastroenteritis worldwide [1]. Major outbreaks have been reported across the United States, Europe, and Asia, reflecting its status as a truly global pathogen [2,3,4]. Since its first identification in 1972, norovirus has been recognized as a major pathogen affecting individuals of all age groups, with transmission commonly occurring through contaminated food and water [5]. Infections are particularly frequent in community and institutional settings such as hospitals, nursing homes, schools, and other confined environments, including commercial and cruise ships [6]. In recent years, increasing attention has been given to natural compounds with antiviral properties. Nevertheless, relatively few studies have investigated natural substances with activity against norovirus [7,8]. Research in this area is complicated by the fact that norovirus cannot be readily propagated in conventional cell culture or animal models, as it requires the human intestine as its host [9]. To address this limitation, *Feline calicivirus* (FCV), a single-stranded RNA virus belonging to the same family, is commonly used as a surrogate model due to its genetic, biochemical, and physicochemical similarities to norovirus [10,11].

Flavonoids, a major class of polyphenolic secondary metabolites, are widely distributed across the plant kingdom [12]. Among them, hesperidin is a prominent flavonoid found abundantly in citrus fruits such as oranges, lemons, and limes. Structurally, it consists of the aglycone hesperetin bound to α-L-rhamnosyl-D-glucose. Extensive research has been conducted on the absorption, bioavailability, and pharmacokinetics of hesperidin and its primary metabolite, hesperetin [13,14]. Hesperidin and its derivatives have been reported to exert diverse pharmacological activities, including anti-inflammatory, antibacterial, antithrombotic, and anticancer effects, as well as inhibitory activity against a range of viruses [15,16,17,18,19,20,21]. However, the poor water solubility of hesperidin limits its practical application in food, cosmetic, and pharmaceutical industries. Various approaches have been explored to enhance its solubility and biological activity. Improved water solubility and antioxidant effects have been reported for hesperidin with chitooligosaccharides [22], while inclusion complexes of hesperidin or hesperetin with hydroxypropyl β-cyclodextrin have been shown to increase solubility and antioxidant potential [23]. Glucosylation of hesperidin has emerged as an efficient and promising approach to increase solubility and bioavailability [24,25].

**Figure 1:**
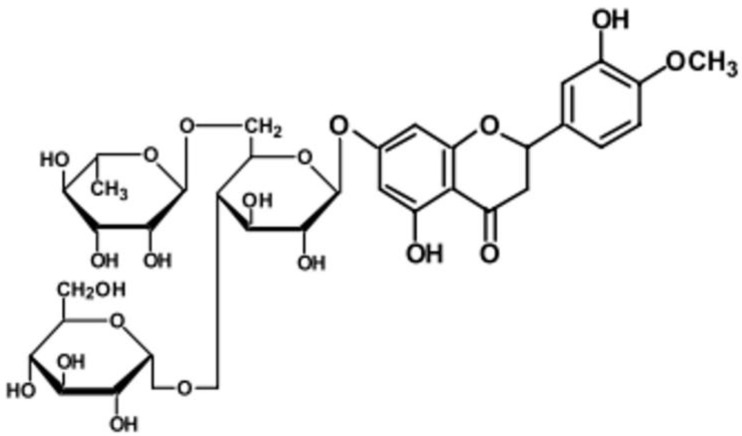
Structure of glucosyl hesperidin (GH).

However, no studies to date have reported on the antiviral effects of glucosyl hesperidin (GH) against FCV or norovirus. In the present study, we investigated the antiviral activity of GH, a water-soluble derivative of hesperidin with reported biological properties [25,26], using FCV as a surrogate model for norovirus. The chemical structure of GH is shown in Figure 1.

## 2. Materials and Methods

### 2.1. Materials

*Feline calicivirus* (FCV, VR-782) and its host cell line, Crandell–Rees feline kidney (CRFK, CCL-94), were obtained from the American Type Culture Collection (ATCC, Manassas, VA, USA). Glucosyl hesperidin (GH) was prepared according to the method described by Choi et al. [25]. Nitazoxanide (Sigma-Aldrich, St. Louis, MO, USA) was used as a positive control in the FCV inhibition assay. Dulbecco’s Modified Eagle’s Medium (DMEM), fetal bovine serum (FBS), phosphate-buffered saline (PBS), trypsin/EDTA, and penicillin–streptomycin solution were purchased from Thermo Fisher Scientific Inc. (Waltham, MA, USA). Reagent WST-1 was purchased from DoGenBio (Seoul, Republic of Korea). All other reagents used were of analytical grade or higher.

### 2.2. Determination of Cytotoxicity

The CRFK cells were cultured in tissue culture flasks using DMEM supplemented with 10% FBS and 1% penicillin/streptomycin at 37 °C in a humidified incubator with 5% CO_2_. When cultures reached approximately 80% confluency, cells were washed with PBS, detached with trypsin/EDTA, and subcultured following centrifugation and resuspension in fresh growth medium. The medium was replaced every 48 h.

To assess the cytotoxicity of GH, cells were seeded into 96-well plates at approximately 60% confluency and incubated for 24 h at 37 °C with 5% CO_2_. The test compound was prepared as a 200 mM stock solution in water and serially diluted in growth medium to final concentrations ranging from 31.2 to 8000 μM. Nitazoxanide was prepared as a 20 mM stock solution in DMSO. Cells were exposed to 40 μL of each test solution for 2 h, after which 100 μL of fresh culture medium was added. Following 24 h of incubation, the test solutions were removed, and cell viability was assessed using the WST-1 assay. Reagent WST-1 was added to each well, and plates were incubated for 30–60 min. Absorbance was measured at 450 nm using a microplate reader. Cell viability was calculated using the following equation.

$$Cell\;viability\left(\%\right)=\frac{Test\;group\;absorbance}{Negative\;control\;group\;absorbance}\;\times 100$$

### 2.3. Determination of Viral Infectivity

Viral infectivity of control and treated samples was determined using a plaque assay as described by Su et al. [27] with minor modifications. Host cells were seeded into 24-well plates at approximately 60% confluency one day prior to infection. Virus stocks were stored at −80 °C, rapidly thawed in a 37 °C water bath, and diluted in virus culture medium to yield an average multiplicity of infection (MOI) of 0.002 per well. After removing the growth medium, cells were inoculated with virus together with GH at final concentrations ranging from 31.2 to 8000 μM (0.2 mL per well). Plates were incubated for 2 h at 37 °C in 5% CO_2_, with intermittent gentle shaking to facilitate uniform virus adsorption. Following incubation, an overlay medium containing low-melting-point agarose was added, and the plates were cultured for 24 h under standard conditions.

At the end of incubation, cells were fixed with 3.5% formaldehyde for 30 min. The fixative was removed, and monolayers were stained with 1% methylene blue for 30 min. Excess stain was washed off with distilled water, and plates were air-dried at room temperature. Plaques, appearing as unstained areas against the stained background, were quantified using ImageJ software (ver. 1.53t, NIH, Bethesda, MD, USA). Viral infectivity titers were expressed as plaque-forming units (PFU) per well. The percentage inhibition of viral infection was calculated using the following equation.

$$Inhibition\;of\;infection\left(\%\right)=\frac{Control\;PFU-Treated\;PFU}{Control\;PFU}\;\times 100$$

The selectivity index (SI) was calculated as the ratio of the 50% cytotoxic concentration (CC_50_) to the 50% inhibitory concentration (IC_50_).

### 2.4. Statistical Analyses

Most experimental procedures were performed in triplicate, and results are expressed as mean ± standard deviation (SD). Statistical analyses were conducted using SPSS software (version 22, SPSS Inc., Chicago, IL, USA). One-way analysis of variance (ANOVA) was applied to compare treatment groups, and significant differences were assessed using Duncan’s multiple range test at levels of *p* < 0.05 and *p* < 0.01.

## 3. Results

### 3.1. Determination of Cytotoxicity

**Figure 2:**
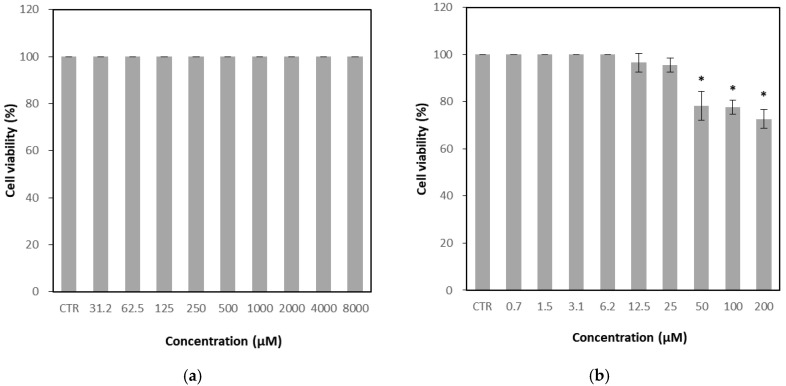
Cytotoxicity of glucosyl hesperidin (GH) and nitazoxanide on Crandell–Rees feline kidney (CRFK) cells. Cells were treated with (**a**) GH or (**b**) nitazoxanide at different concentrations, respectively. Results were represented as mean ± SD (*n* = 3). Significant differences from the control (* *p* < 0.05). Note: CTR, Control.

The CRFK cell line, derived from feline kidney epithelium, is highly susceptible to FCV infection and was therefore used as the host cell for antiviral evaluation. The cytotoxicity of GH was assessed by two-fold serial dilutions up to a maximum concentration of 8000 μM and compared with untreated control cells. No significant cytotoxic effects were observed at any concentration tested, with cell viability maintained at 100% even at 8000 μM (Figure 2a). Accordingly, the 50% cytotoxic concentration (CC_50_) of GH could not be precisely determined and was estimated to be greater than 8000 μM. In contrast, the positive control, nitazoxanide, exhibited marked cytotoxicity, reducing cell viability to 78.2% at 50 μM (Figure 2b).

### 3.2. Determination of Anti-Viral Activity

The infectivity titer of the virus was determined by performing 10-fold serial dilutions of the viral stock, followed by plaque counting after 24 h of infection in host cells. The infectivity titer (PFU/mL) was calculated using the following equation.

$$Infectivity\;titer\;(PFU/mL)=\frac{Number\;of\;plaques\left(PFU\right)\times Dilution\;factor}{Volume\;of\;virus\;added\;(mL)}\;\;$$

**Figure 3:**
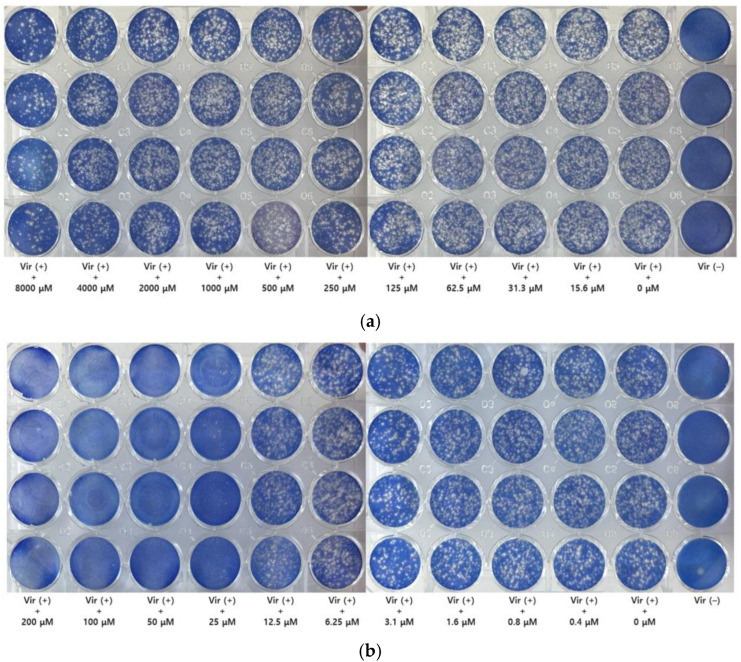
Plaque assay for glucosyl hesperidin (GH) and nitazoxanide. Plaque assay was performed to evaluate the antiviral activity of GH. The experiment was conducted at a low multiplicity of infection (MOI = 0.002). Cells were treated with (**a**) GH or (**b**) nitazoxanide at different concentrations, respectively.

Based on the calculated infectivity titer, the viral stock was diluted to achieve a multiplicity of infection (MOI) of approximately 0.002 for the main assay. The results of the plaque reduction assay with GH at this MOI are presented in Figure 3. Untreated cells (without GH treatment) served as a reference, which functionally represented the negative control.

**Figure 4:**
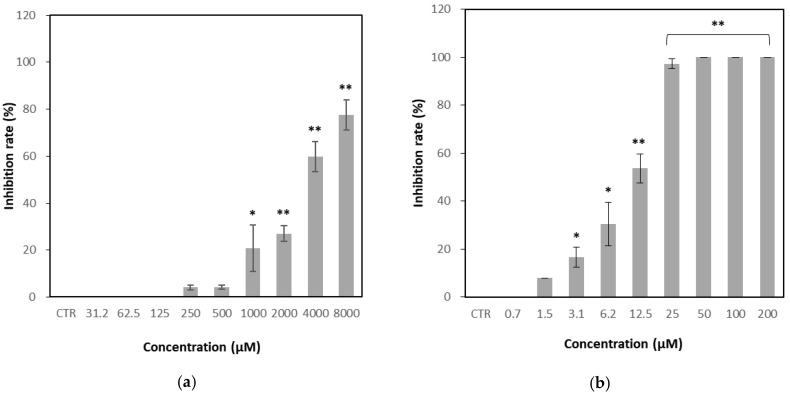
Effect of glucosyl hesperidin (GH) on viral infection inhibition rate. Crandell–Rees feline kidney (CRFK) cells were treated with (**a**) GH or (**b**) nitazoxanide at different concentrations, respectively. Results were represented as mean ± SD (*n* = 3). Significant differences from the control (* *p* < 0.05, ** *p* < 0.01). Note: CTR, Control.

As shown in Figure 4, GH exhibited concentration-dependent antiviral activity against FCV, with an inhibition rate of 4.1% at 250 μM and 77.6% at the maximum tested concentration of 8000 μM. The calculated IC_50_ value for GH was 3281 μM, whereas that of the positive control, nitazoxanide, was 9.6 μM.

**Figure 5:**
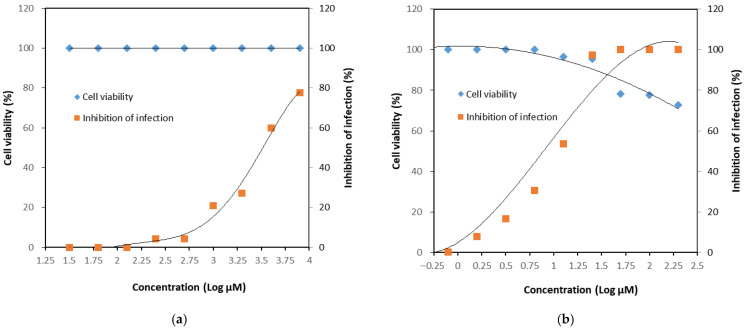
Dose–response relationship of viral infection inhibition. Crandell–Rees feline kidney (CRFK) cells were treated with (**a**) glucosyl hesperidin or (**b**) nitazoxanide at different concentrations, respectively.

Although a definitive CC_50_ value could not be established, the dose–response relationship was assessed (Figure 5). For GH, no cytotoxicity was observed in CRFK cells within the tested concentration range, and the CC_50_ was estimated to be at least 8000 μM. Based on the IC_50_ value of 3281 μM, the selectivity index (SI) was calculated to be greater than 2.44. In comparison, the positive control nitazoxanide exhibited a CC_50_ value exceeding 200 μM, an IC_50_ of 9.6 μM, and a corresponding SI greater than 20.81.

## 4. Discussion

Many natural products, including plant extracts, flavonoids, essential oils, and their derivatives, have been investigated for their anti-norovirus activity using FCV as a surrogate model [28,29,30,31,32]. Flavonoids, in particular, are recognized as promising antiviral agents against both respiratory and enteric viruses [33,34]. Their mechanisms of action include direct inhibition of viral proteases, interference with viral RNA synthesis, and modulation of host immune responses [25]. Hesperidin and its aglycone, hesperetin, have been reported to inhibit SARS-CoV-2 spike-mediated syncytial formation, thereby blocking cell-to-cell viral spread [33]. In addition, hesperetin has been shown to suppress chikungunya virus replication and reduce rotavirus infection, supporting its broad antiviral activity against RNA viruses, including enteric pathogens [34]. Beyond direct antiviral and antibacterial effects, hesperidin-type compounds also promote cellular recovery through antioxidant and anti-inflammatory activities [35,36,37]. Nevertheless, the precise inhibitory concentrations of hesperidin-related compounds against norovirus or FCV have rarely been established, and most studies remain limited in scope [38]. Furthermore, the poor water solubility of hesperidin restricts its broader application.

In the present study, GH, a water-soluble derivative of hesperidin, exhibited measurable antiviral activity against FCV while maintaining minimal cytotoxicity in CRFK cells. Previous studies have reported lower cytotoxicity of GH compared with hesperidin in RAW 264.7 and HaCaT cells [25,26]; the results of this study extend these observations by confirming its safety profile in CRFK cells. Cytotoxicity assays demonstrated that GH maintained 100% cell viability even at the maximum tested concentration of 8000 μM, whereas the reference antiviral nitazoxanide induced cytotoxicity at 50 μM. Antiviral assays further showed that GH inhibited FCV replication in a concentration-dependent manner, with an IC_50_ of 3281 μM. Although glucosyl hesperidin exhibited a relatively low SI value, its negligible cytotoxicity suggests potential advantages in terms of safety and tolerability. The high CC_50_ value (>8000 μM) further underscores its safety margin, a key consideration in the development of antivirals for long-term treatment or prophylactic use. Importantly, the balance between solubility, antiviral efficacy, and cytotoxicity is critical for developing effective therapeutic agents. Structural modifications of flavonoids, such as glycosylation or esterification, have been shown to enhance their biological performance [25,26,39,40]. Viral inhibition can occur through mechanisms such as interference with adsorption or penetration, suppression of viral replication, or enhancement of host cell recovery, depending on the specific compound [41,42]. Flavonoids have also been reported to inhibit viral infection through mechanisms that vary according to their structural characteristics [8]. Further studies are required to elucidate the mechanism of action of GH.

While higher SI values are typically regarded as desirable in evaluating antiviral agents, a definitive threshold for this evaluation has not been established. The selectivity index (SI) values of natural products can be influenced by factors such as the viral strain, the chemical structure of the compounds, and the assay conditions used in the experiments [38,43]. The SI value of GH was over 2.44 in this study, which is generally considered insufficiently effective, but for natural products, it may be considered to have potential for development and may require further investigation. It is also suggested that lower SI values may confer certain benefits, where they are often associated with enhanced water solubility and, consequently, improved bioavailability.

Glucosylation of hesperidin enhances hydrophilicity and improves solubility in aqueous media, thereby facilitating the use of higher effective dosages. Considering these properties, further investigation into the structure–activity relationship is warranted to optimize its antiviral efficacy and fully exploit its potential as a safe and effective therapeutic agent.

## 5. Conclusions

To evaluate the applicability of GH, a highly water-soluble derivative of hesperidin, its antiviral activity against FCV was assessed. It exhibited no detectable cytotoxicity in CRFK cells at concentrations up to 8000 μM and inhibited FCV replication in a concentration-dependent manner, with an IC_50_ value of 3281 μM. To our knowledge, this is the first study to demonstrate the antiviral activity of GH against FCV, a surrogate for human norovirus. These findings suggest that GH has potential for broader applications as a safe, naturally derived antiviral agent.

## Data Availability

The original contributions presented in this study are included in the article. Further inquiries can be directed to the corresponding author.

## Abbreviations

The following abbreviations are used in this manuscript:

GH	Glucosyl hesperidin
FCV	Feline calicivirus
CRFK	Crandell–Rees feline kidney
PFU	Plaque-forming units
SI	Selectivity index
CC_50_	50% cytotoxic concentration
IC_50_	50% inhibitory concentration
MOI	Multiplicity of infection
